# Dynamic Active Sites in Electrocatalysis

**DOI:** 10.1002/anie.202415794

**Published:** 2024-10-31

**Authors:** Minghui Ning, Sangni Wang, Jun Wan, Zichao Xi, Qiao Chen, Yuanmiao Sun, Hui Li, Tianyi Ma, Huanyu Jin

**Affiliations:** ^1^ Institute of Technology for Carbon Neutrality Shenzhen Institute of Advanced Technology Chinese Academy of Sciences Shenzhen 518055 Guangdong China; ^2^ Centre for Atomaterials and Nanomanufacturing (CAN) School of Science RMIT University Melbourne VIC-3000 Australia; ^3^ State Key Laboratory of New Textile Materials and Advanced Processing Technologies Hubei Key Laboratory of Biomass Fibers and Eco-Dyeing & Finishing Wuhan Textile University Wuhan 430200 Hubei China; ^4^ ARC Industrial Transformation Research Hub for Intelligent Energy Efficiency in Future Protected Cropping (E2Crop) Melbourne VIC-3000 Australia

**Keywords:** dynamic active sites, electrocatalysis, in situ*/*operando characterization, dynamic reconstruction, structure–activity correlations

## Abstract

In‐depth understanding of the real‐time behaviors of active sites during electrocatalysis is essential for the advancement of sustainable energy conversion. Recently, the concept of dynamic active sites has been recognized as a potent approach for creating self‐adaptive electrocatalysts that can address a variety of electrocatalytic reactions, outperforming traditional electrocatalysts with static active sites. Nonetheless, the comprehension of the underlying principles that guide the engineering of dynamic active sites is presently insufficient. In this review, we systematically analyze the fundamentals of dynamic active sites for electrocatalysis and consider important future directions for this emerging field. We reveal that dynamic behaviors and reversibility are two crucial factors that influence electrocatalytic performance. By reviewing recent advances in dynamic active sites, we conclude that implementing dynamic electrocatalysis through variable reaction environments, correlating the model of dynamic evolution with catalytic properties, and developing localized and ultrafast in situ*/*operando techniques are keys to designing high‐performance dynamic electrocatalysts. This review paves the way to the development of the next‐generation electrocatalyst and the universal theory for both dynamic and static active sites.

## Graphical abstract

The dynamic evolutions of active sites are ubiquitous in electrocatalysis due to the applied potentials and reaction environments. The study on dynamic active sites deciphers the intrinsic structure–property relationships and provides more comprehensive insights into the interaction between electrocatalysts and electrochemical reactions.

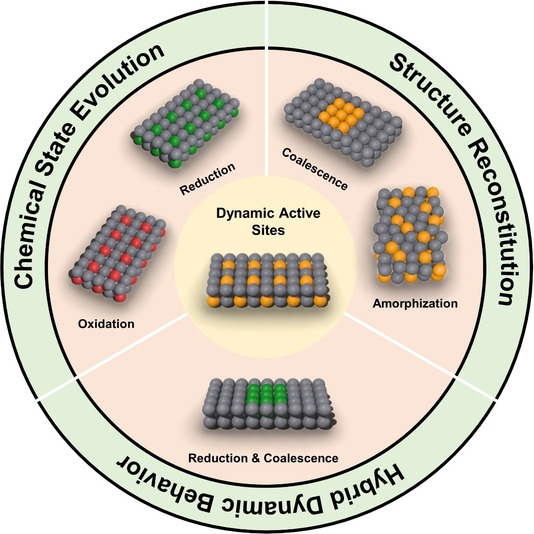

## 1 Introduction

Electrocatalysis is an important subject related to clean energy production, green chemical industry, and environmental governance. In electrocatalysis, an active site can be an atom or a group of atoms that accelerate the reaction kinetics of an electrochemical process.^[1]^ Different from other types of chemical catalysis, electrocatalysis is a complex multiphase and mostly dynamic process involving the interactions between catalyst, electrolyte, potential, reactant, and product.[^2^, ^3^, ^4^] Due to such interactions, the pristine state of the active sites might not be able to maintain, and dynamic changes might happen to the active sites and play a critical role in their activities during the electrocatalysis.[^5^, ^6^, ^7^] Therefore, deeper insights into the dynamic evolution of active sites are highly required for the further development of electrocatalysis.

Traditionally, active sites are regarded as static models with fixed structural and electronic properties during electrocatalysis, which is well studied for some highly stable electrocatalysts in certain electrochemical reactions, such as Pt (111) for hydrogen production.[^8^, ^9^] However, such static point of view cannot fully elucidate electrocatalysis on nanomaterials, and recent studies unveiled that many electrocatalysts experienced dynamic evolution during electrochemical processes.[^10^, ^11^] Dynamic active sites in the context of electrocatalysis refers to the active regions on a catalyst's surface that undergo geometric or electronic structural changes in response to the varying environment conditions, reactants, and applied electrochemical potentials.^[12]^ Different from static active sites with fixed geometric and electronic structures, the dynamic active sites with variable/adaptive geometric and electronic structures exhibit better adaptability to the electrocatalysis process.[^13^, ^14^] Such adaptability exhibits variable catalytic property to fit the complex requirements of the activity, stability, and selectivity to the complex multi‐electron reactions such as hydrogen evolution reaction (HER), oxygen evolution reaction (OER), carbon dioxide reduction reaction (CO_2_RR), oxygen reduction reaction (ORR), hydrogen oxidation reaction (HOR), nitrogen reduction reaction (NRR), etc.[^2^, ^15^, ^16^, ^17^, ^18^, ^19^, ^20^] The design of dynamic active sites is crucial for developing effective, selective, and durable electrocatalysts, which are essential for the widespread application of sustainable energy technologies. This capability allows for tuning catalytic properties during reactions to meet specific requirements or to overcome common challenges such as catalyst degradation, poisoning, or low activity under varying operating conditions.

The unique characteristics promoted the rapid development of dynamic active sites. For example, the chemical state evolution of dynamic active sites has been widely reported in multiple fields.[^14^, ^21^, ^22^] The valence oxidation of transition metal active sites is attributed to the origin of their high activity for OER, while the valence reduction of Cu sites is correlated to their activity and selectivity towards CO_2_RR.[^7^, ^14^] Besides, the structure reconstitution is ubiquitous during dynamic evolution of active sites.[^6^, ^23^] The coalescence of single atoms was reported in many reductive electrochemical reactions such as CO_2_RR and ORR, during which the coordination environment and thus the catalytic property were significantly altered. The surface amorphization is usually accompanied with the generation of real active species during dynamic electrocatalysis. To study the dynamic active sites, the advanced in situ*/*operando characterizations have been developed to observe the dynamic behaviors and reaction intermediates. The in situ spectroscopy such as X‐ray absorption spectroscopy (XAS) can be employed to monitor the chemical state and coordination environment changes, while the in situ microscopy such as in situ transmission electron microscopy (TEM) can be applied to investigate local structure reconstitution, and in situ mass spectroscopy is advantageous in directly probing the reaction intermediates.[^24^, ^25^] Previous reviews have carried out discussions upon dynamic evolution behaviors, electrochemical reactions, or a certain type of dynamic active site. However, a comprehensive understanding of dynamic active sites is left to be summarized.[^2^, ^3^, ^10^] Therefore, an in‐depth understanding of how the dynamic evolution influences activity, stability, and selectivity of active sites is needed.

This review presents a thorough discussion of the dynamic active sites for various electrocatalytic reactions. The primary objective of this review is to investigate the most concern aspects of dynamic active sites, particularly focusing on the dynamic behaviors, reversibility, and the characterization techniques. Firstly, by presenting a systematic comparison between static and dynamic active sites, we unveil the ubiquitous and versatile natures of dynamic active sites, and their superior catalytic property granted by the dynamic electronic and geometric structures. Secondly, we reveal the structure‐property correlation of dynamic active sites via summarizing the dynamic behaviors including chemical state evolution, structure reconstitution, and hybrid dynamic behavior. Thirdly, we discover the thermodynamics and kinetics of the reversed evolution determine the reversibility of the dynamic evolution, and the reversibility strongly correlates with the stability of dynamic active sites. Fourthly, we propose a combination of the advanced in situ*/*operando techniques should be rationally adopted from the perspective of their own advantages and limitations. Then, the efficient protocols for characterizing the evolution behavior and optimizing the performance are discussed by overviewing the recent advances of dynamic active sites. Finally, future perspectives are outlined to provide important insights into the challenges and opportunities of dynamic active sites.

## 2 Dynamic vs. Static Active Sites

**Figure 1:**
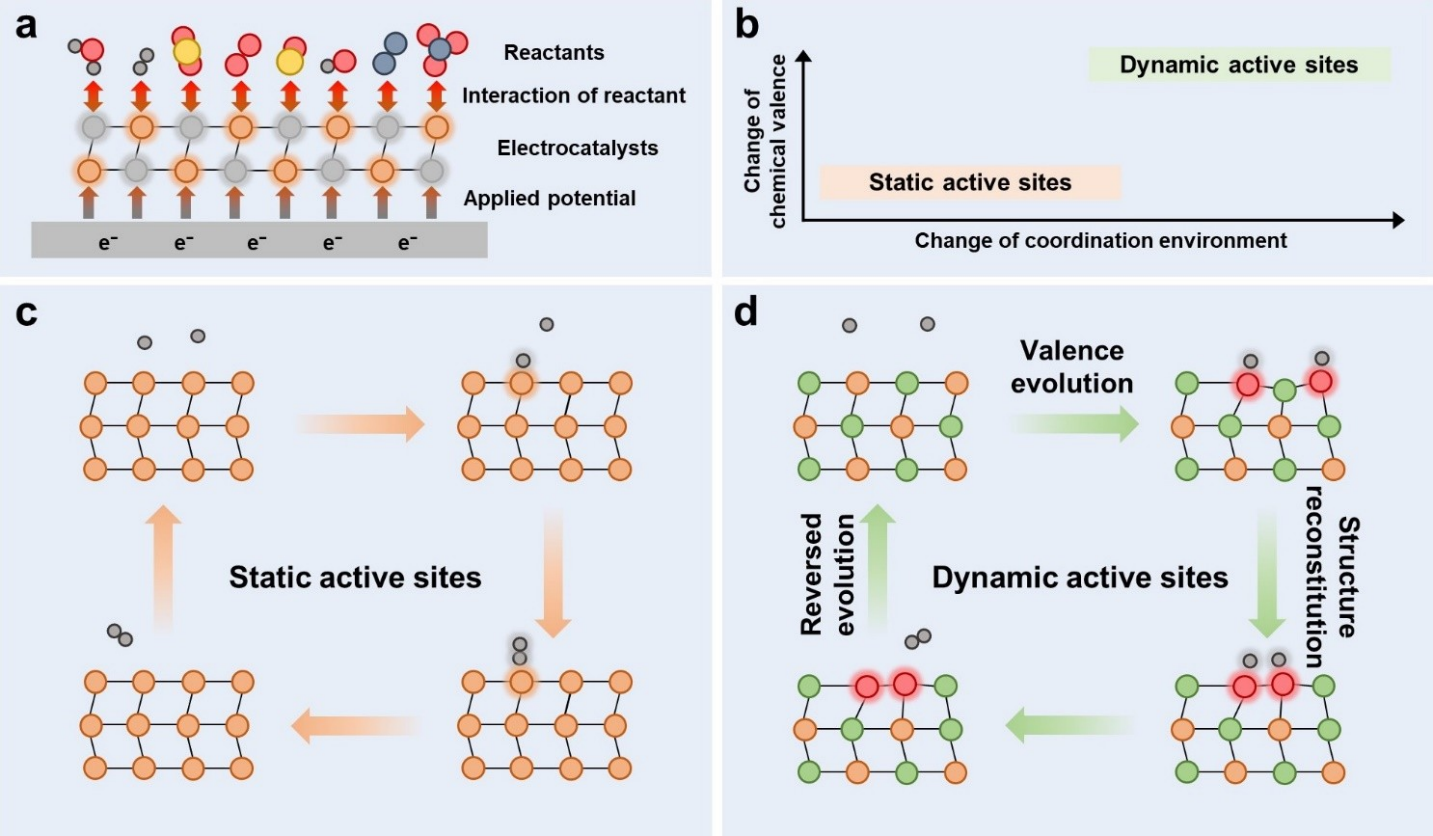
Comparison between dynamic and static active sites. (a) The influences of applied potential and reaction environment on the dynamic active sites. (b) Change of chemical valence and coordination environment of static and dynamic active sites during electrocatalysis. (c and d) Typical evolution process of static and dynamic active sites during electrocatalysis, respectively.

Active sites refer to specific surface sites on a catalyst that possess high catalytic activity, accelerating the reaction kinetics of a given chemical reaction.^[1]^ Conventionally, many studies considered active sites with fixed electronic and geometric structures as static and non‐interacting models during electrocatalysis.[^26^, ^27^, ^28^, ^29^] Although many theoretical studies have involved the applied potential and transition state into the simulation and successfully predict new electrocatalysts in multiple fields, the interactions between active sites, reaction intermediates and reaction environments remain largely neglected.[^30^, ^31^] In practice, the equilibrium geometry of electrocatalyst is not guaranteed in a electrochemical process, during which the reaction environment, applied potential, and reactant might change the geometric, electronic structures, and thus the catalytic properties of electrocatalysts (Figure 1a). Yet, an increasing amount of research reports the dynamic evolution of electrocatalysts during the heterogeneous electrocatalysis process.[^2^, ^3^]

Static active sites reside in the highly stable electrocatalysts that can withstand the influence from reaction environments, applied potentials, and remain unchanged during electrocatalysis (Figure 1b). A classic instance of static active sites is Pt single crystal for HER, in which one proton is adsorbed on the surface of the Pt atom, followed by the subsequent adsorption of another proton to produce a H_2_ molecule (Figure 1c).[^9^, ^32^] As static active sites, the Pt atoms maintained their geometric and electronic structure during the HER process. In contrast, dynamic active sites originate from the instability of catalysts in electrochemical reactions, during which the electronic and geometric structures thus the catalytic properties experience dynamic evolution. For examples, the coalescence of two active sites with H_ads_ can facilitate the recombination of two H_ads_ to produce H_2_ molecule, switching the Volmer–Heyrovsky mechanism to the kinetically faster Volmer–Tafel mechanism (Figure 1d).^[33]^ Besides, the M−N−C materials are susceptible to the reactant/intermediate adsorption and applied potential, and the dynamic evolution of the M‐N_x_ moiety may happen during the electrochemical reactions.[^34^, ^35^] Highly reversible chemical valence state variations can be observed on transition metal oxyhydroxides during the alkaline OER process.[^36^, ^37^] And the lattice oxygen‐mediated mechanism (LOM) provides direct evidence that the dynamic active sites can be part of the electrochemical reaction.[^38^, ^39^] In CO_2_RR, the dynamic evolution of the chemical valence state of Cu active site is a common phenomenon and has strong relation with the activity and selectivity of the reaction.[^2^, ^40^] The group 3–4 elements such as Sc, Y, La, Ti, Zr, and Hf, can easily lose their outermost *s* and *d* electrons due to their strong metal‐ligand covalency bonding, which is stable but typically inactive to the oxygen involved reactions.^[41]^ But in a YN_4_ motif with *d*‐electron deficient Y single atom, the remaining *d* electron can be bounded with an axial ligand X. The YN_4_−X covalency can adaptively contribute a certain amount of electron to dynamically bind with different intermediates, triggering a Pt like activity for ORR on the inactive YN_4_.^[41]^ During the electrochemical reduction of nitrate to ammonia process, the Cu−N_4_ single atom catalyst (SAC) underwent the transformation from Cu−N_4_ to Cu−N_3_, then the aggregation of Cu^0^ single atoms to Cu nanoparticles, with the applied potential moving from 0.00 V to −1.00 V vs. RHE.^[42]^ The dynamic evolution of active sites during electrocatalysis is ubiquitous in many catalysts and reactions, so the in‐depth understanding of the structure–activity correlations during dynamic evolution are of great significance to the further development of electrocatalysis.

## 3 Dynamic Behavior During Catalytic Reactions

Numerous literatures have been reported with different kind of dynamic behaviors including valence oscillation, chemical state oxidation or reduction, defect formation, atomic coalescence, element dissolution and precipitation, morphology reconstruction, etc.[^2^, ^3^, ^5^, ^13^, ^43^, ^44^] Although the different dynamic behaviors seem to be irrelevant, they can be mainly categorized into two types of behavior that are 1) chemical state evolution and 2) structure reconstitution from the perspective and electronic and geometric dynamic evolution.[^2^, ^6^, ^14^] Notably, the different dynamic behaviors can be entangled and happened simultaneously.[^45^, ^46^, ^47^, ^48^] Very often, one kind of dynamic behavior might drive or accelerate another kind of dynamic behavior during electrocatalysis. In this section, the dynamic behaviors are first systematically overviewed based on the two categories of chemical state evolution and structure reconstitution, then the hybrid dynamic behaviors are discussed to unveil the entanglement between different dynamic evolution processes.

### 3.1 Chemical State Evolution

Metal elements especially for the multi‐valence metal elements could typically experience chemical state evolution during electrocatalysis, since the redox potential of different valence is within the working potential window.[^14^, ^36^, ^49^] The evolution of valence state changes the electronic structure of the active sites and the adsorption energy to reaction intermediates. According to different evolution directions, the chemical state evolution can be further differentiated into valence oxidation, valence reduction, and valence oscillation. In an oxidation electrochemical reaction, the valence state of dynamic active site tends to increase due to the applied oxidative potential and the electron withdrawing nature of the reaction.^[50]^ Conversely, the valence state of dynamic active site tends to decrease in a reduction electrochemical reaction.[^17^, ^51^] Both the valence oxidation and reduction are related to the electronic structure and redox potential of dynamic active site, but mainly driven by the applied potential and reaction environment. Differently, the chemical valence state of dynamic active site undergoes reciprocal changes for valence oscillation. Though the valence oscillation is powered by the applied potential in general, its reciprocal changes mainly originated from the interaction between different elements inside the dynamic active sites. The following discussions systematically elaborate the dynamic processes of different chemical state evolutions and how they affect the catalytic activity, stability, and selectivity of dynamic active sites.

**Figure 2:**
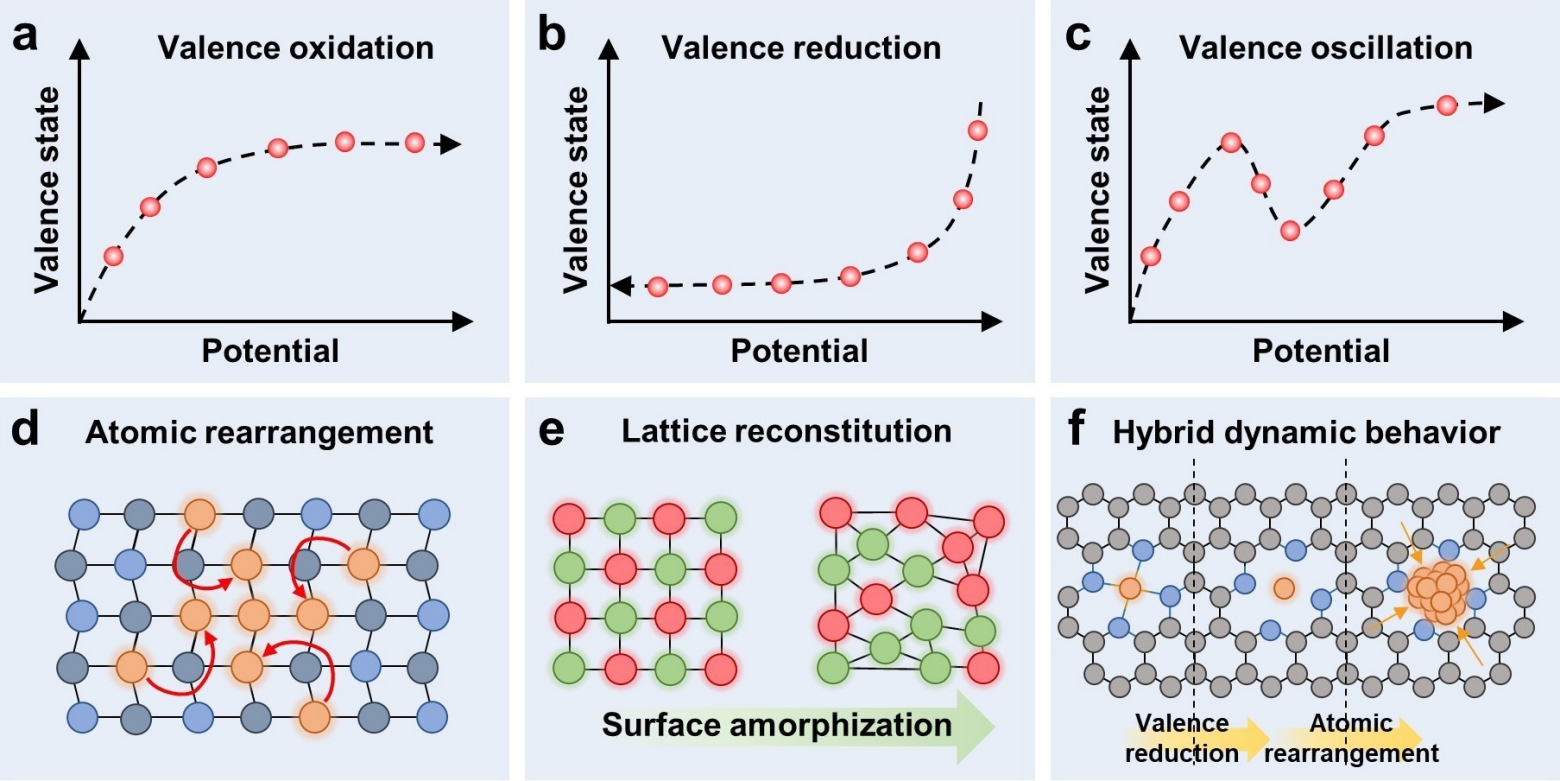
Different types of dynamic evolution behaviors of dynamic active sites. (a–c) Valence oxidation, valence reduction, and valence oscillation of chemical state evolution, respectively. (d and e) Atomic rearrangement and lattice reconstitution of structure reconstitution, respectively. (f) Hybrid dynamic behavior.

The valence oxidation is an ubiquitous dynamic evolution behavior in oxidation electrochemical reactions like OER.[^50^, ^52^] Fe, Co, and Ni are the frequently studied elements for alkaline OER, and their redox potentials (for examples, Ni(II)/Ni(III), Fe(III)/Fe(IV), Co(III)/Co(IV)) are located within the OER working potential windows.[^37^, ^53^] During the OER process, these metal elements will typically be first oxidized to higher valence state (Figure 2a), then the high valence transition metal sites will play as the real active species during the reaction. Moysiadou et al. reported that the dynamic valence oxidation of CoOOH during water oxidation.^[54]^ The pristine Co(III)OOH mixed with some Co(II) was slightly oxidized after immersed in the KOH electrolyte, which could be attributed to the oxidation of Co(II) to Co(III) by the OH^−^. In situ XAS results revealed the valence state of Co increased slightly with the increased applied potential. When the potential was higher than 1.45 V, the formation of Co(IV) produced the Co‐superoxide intermediate. Eventually, the combination of two lattice oxygen from the Co‐superoxide released the O_2_ molecule.

In the reduction electrochemical reactions such as CO_2_RR, HER, ORR, and NRR, the valence state reduction of active site is frequently observed during the electrocatalysis process (Figure 2b).[^2^, ^16^, ^17^, ^51^, ^55^] In CO_2_RR, Cu based electrocatalysts are the most studied materials due to their excellent activity and highly tunable selectivity.^[56]^ Thermodynamically, the Cu based catalysts can be reduced to Cu^0^ under the working potential of CO_2_RR due to the redox potential of Cu^+^/Cu^0^ (−0.36 V vs. NHE).[^14^, ^56^] However, not all Cu materials can be completely converted to Cu^0^. The reoxidation of Cu^0^ and slow kinetics of Cu^+^→Cu^0^ can lead to an equilibrium of Cu^+^/Cu^0^ conversion, allowing the existence of Cu^+^ during CO_2_RR. The different valence state of Cu species can lead to different selectivity to the products.^[14]^ The Cu^+^ active sites have relatively weak adsorption energy to *CO adsorbates, and thus lead to the desorption of *CO to generate CO/HCOO^−^. The Cu^0^ active sites process stronger adsorption energy to *CO intermediates, and thereby further hydrogenation can proceed to produce CH_4_. It was also reported that the mixture of Cu^0^ and Cu^+^ active sites would increase the selectivity of C_2_ product in CO_2_RR.[^57^, ^58^] Therefore, the valence reduction of active sites is mainly driven by the cathodic potential in electrochemical reduction reactions, which can tune the electronic structure and thus alter the selectivity of the reaction like CO_2_RR.

Similar to the valence oxidation and reduction, the valence oscillation is generally powered by the applied potential. Due to the geometric and electronic interaction within the catalyst, the valence evolution of active sites is not consistent but exhibits reciprocating feature during the electrochemical process (Figure 2c).[^59^, ^60^, ^61^] Kang et al. developed a monolayer NiCo hydroxide via doping the Co into the monolayer Ni(OH)_2_.^[61]^ The unoccupied *d_x_* ^*2*^_*–y*_^*2*^ orbital of Co(III) was lower than Ni(III), and the Co(III) was prone to accept the excess electrons from NiCo hydroxide. Hence, the doped Co(III)/Co(IV) is prone to be reduced to lower valence state compared to the Ni(III)/Ni(IV), thus promoting the formation of O vacancy (O_V_). In situ X‐ray absorption fine structure (XAFS) showed the valence states of Ni and Co sites experienced an obvious oscillation with increased applied potential, which can be attributed to the formation of O_V_. The electrochemical results together with in situ XAFS suggested that the formation of O_v_ and the valence state oscillation dynamically generated the active sites for OER. Deng et al. reported that the introduction of high‐valent W species triggered the valence oscillation of Ru in RuO_x_.^[59]^ The electrons shuttled between the Ru and W sites inside the Ru−O−W functional units, which weakened the adsorption energies for oxygen intermediates and prevented the over‐oxidation and dissolution of Ru sites during acidic water oxidation. Besides, Jin et al. reported a Re_0.06_Ru_0.94_O_2_ for efficient and stable acidic oxygen evolution.^[60]^ The Re atoms adaptively accepted electron to activate the Ru sites at on‐site potential, then donated electron to prevent the over‐oxidation and dissolution of Ru sites at large overpotential. Hence, rational valence oscillation can adaptively activate and stabilize the dynamic active sites, thus achieving exceptional performance in electrocatalysis.

### 3.2 Structure Reconstitution

The geometric structure is closely related to the catalytic property of electrocatalyst.[^62^, ^63^] The dynamic structure reconstitution can reshape the geometric structure and change the catalytic nature of electrocatalyst. Hence, the principles of dynamic structure reconstitution are of great significance for unveiling the real active species and guiding the rational design of electrocatalysts. The structure reconstitution can be further differentiated into atomic rearrangement and lattice reconstitution. In this following discussion, the dynamic structure reconstitution will be thoroughly discussed to unveil the nature of atomic rearrangement and lattice reconstitution.

Atomic rearrangement refers to the reconstruction of a specific type of active site (typically atom) without changing the bulk material significantly (Figure 2d). Such atomic rearrangement only leads to the dynamic evolution of the electronic and geometric structure of active sites, while the property of bulk materials remains nearly unchanged during electrocatalysis. The atomic rearrangement typically happens on the catalyst with low content of active site and highly stable bulk material.[^42^, ^64^, ^65^] SAC is a typical species that tends to undergo atomic rearrangement. Wei et al. prepared a copper single atoms decorated CeO_2_ (Cu_1_−CeO_2_) for urea synthesis.^[64]^ In situ XAS showed that the single atom Cu_1_ dynamically evolved to the nano cluster Cu_4_, in which the Cu_4_ facilitated the C−N coupling and promoted the urea formation. Yang et al. reported the dynamic structural evolution from a Cu−N_4_ single‐atom site to a ~5 nm Cu nanoparticle during nitrate reduction to ammonia.^[42]^ DFT simulations illustrated the excellent activity of the Cu nanoparticle under −1.00 V vs. RHE for nitrate reduction to ammonia. Further experiments revealed that such coalescence is reversible, in which the nanoparticle disintegrated into the Cu−N_4_ single atom after exposing to an ambient atmosphere.

Lattice reconstitution is another typical type of structure reconstitution.[^6^, ^7^, ^66^] Different from atomic rearrangement, the bulk material undergoes a significant lattice change during lattice reconstitution (Figure 2e), thus leading to the alteration of catalytic property. Surface reconstruction is a typical lattice reconstitution, which has been widely studied in water oxidation.[^7^, ^67^] Wei et al. substituted Al in La_0.3_Sr_0.7_CoO_3–δ_ (LSCAO‐0.2) to promote the surface reconstruction from the oxide to the active oxyhydroxide and activate the lattice oxygen as an active site.^[68]^ The high‐resolution TEM (HRTEM) revealed the surface lattice facet of LSCAO‐0.2 changed from (110) to (200). It is very interesting that such surface reconstruction was terminated after the leaching of the surface Al element, which stabilized the overall structure and maintained the activity of LSCAO‐0.2. More often, the leaching or dissolution of element will initiate the lattice reconstitution of electrocatalyst. An example is the leaching of V accelerated the reconstruction of Fe‐modified Co_2_VO_4_, forming a highly active (Fe,V)‐doped CoOOH for OER.^[69]^ The remaining V in the form of V_2_O_3_ provided dynamic charge compensation and prevented the excessive loss of lattice oxygen and Co dissolution.

### 3.3 Hybrid Dynamic Behavior

The chemical valence evolution and structure reconstitution are highly entangled during the dynamic evolution of active sites.[^2^, ^3^] The evolution of chemical valence will change the coordination number and environment, which might induce the geometric reconstitution of electrocatalyst. On the other hand, the structure reconstitution will reshape the chemical environment of electrocatalyst, leading to the chemical valence changes. In the following, the hybrid dynamic behaviors of active sites are summarized, and the unique significance of hybrid dynamic evolution is addressed.

Cu−N−C is a classic active sites for many electrochemical reduction reaction such as CO_2_RR, ORR, and NRR.^[70]^ Under the cathodic potential, the breaking of C−N bonds produces the metallic state Cu^0^, accompanying with the coalescence of Cu^0^ single atoms (Figure 2f).[^42^, ^65^] Thus, the valence reduction of Cu−N−C is highly entangled with its atomic rearrangement. Ning et al. designed an Fe doped Ni&Ni_0.2_Mo_0.8_N for highly efficient bifunctional alkaline water/seawater electrolysis based on electrochemical reconstruction.^[71]^ Under the anodic potential and alkaline electrolyte, the Fe doped Ni&Ni_0.2_Mo_0.8_N underwent the valence oxidation accompanying with Mo and N dissolution, eventually reconstructed into the Fe and Mo co‐doped NiO. With the pristine Fe doped Ni&Ni_0.2_Mo_0.8_N as highly efficient HER catalyst, and the in situ reconstructed Fe and Mo co‐doped NiO as efficient OER catalyst, the Fe doped Ni&Ni_0.2_Mo_0.8_N||Fe and Mo co‐doped NiO electrolyzer exhibited the state‐of‐art bifunctional alkaline water/seawater electrolysis performance. Furthermore, Zhao et al. developed a Fe‐doped cobalt phosphide nanoboxes (Co@CoFe−P NBs) for bifunctional water electrolysis, which can dynamically evolved towards both the HER and OER active sites.^[72]^ Under the HER working condition, the partial Fe substitution promoted the structural reconstruction of the HER active P−Co−O−Fe−P alongside with the valence reduction. While under the OER working condition, the reconstructed Co^IV^−O−Fe^IV^ moieties evolved via atomic rearrangement and valence oxidation exhibited excellent OER activity. Therefore, the hybrid dynamic evolution is capable of completely reshaping the electronic and geometric properties of electrocatalyst, thus bringing unexpected outcomes, especially in multi‐functional electrocatalysis.

The valence state evolution can alter the electronic structure while the structure reconstitution can change the geometric structure of the electrocatalysts. One type of dynamic behavior might accompany with or even trigger another type of dynamic behavior, leading to the hybrid dynamic behavior. The change of electronic and geometric structure can optimize the adsorption energies for the reaction intermediates and stabilize the active species under harsh condition, achieving better activity, stability and selectivity. Therefore, the dynamic behaviors can significantly improve the catalytic performance via changing the electronic and geometric structure of electrocatalysts.

## 4 Reversibility and Stability of Dynamic Active Sites

The dynamic evolution behaviors of electrocatalysts have been intensively investigated and in‐depth understanding of structure–activity relationship of dynamic active sites has been discussed.[^2^, ^3^, ^62^, ^63^] However, the reversibility and stability of the dynamically evolved active sites have not been thoroughly discussed before and remained relatively ambiguous. In this section, the reversibility of the dynamic evolution process is first discussed from the perspectives of thermodynamics and kinetics, and the stability of dynamic active sites is systematically overviewed to unveil its relationship with the reversibility.

### 4.1 Reversibility

**Figure 3:**
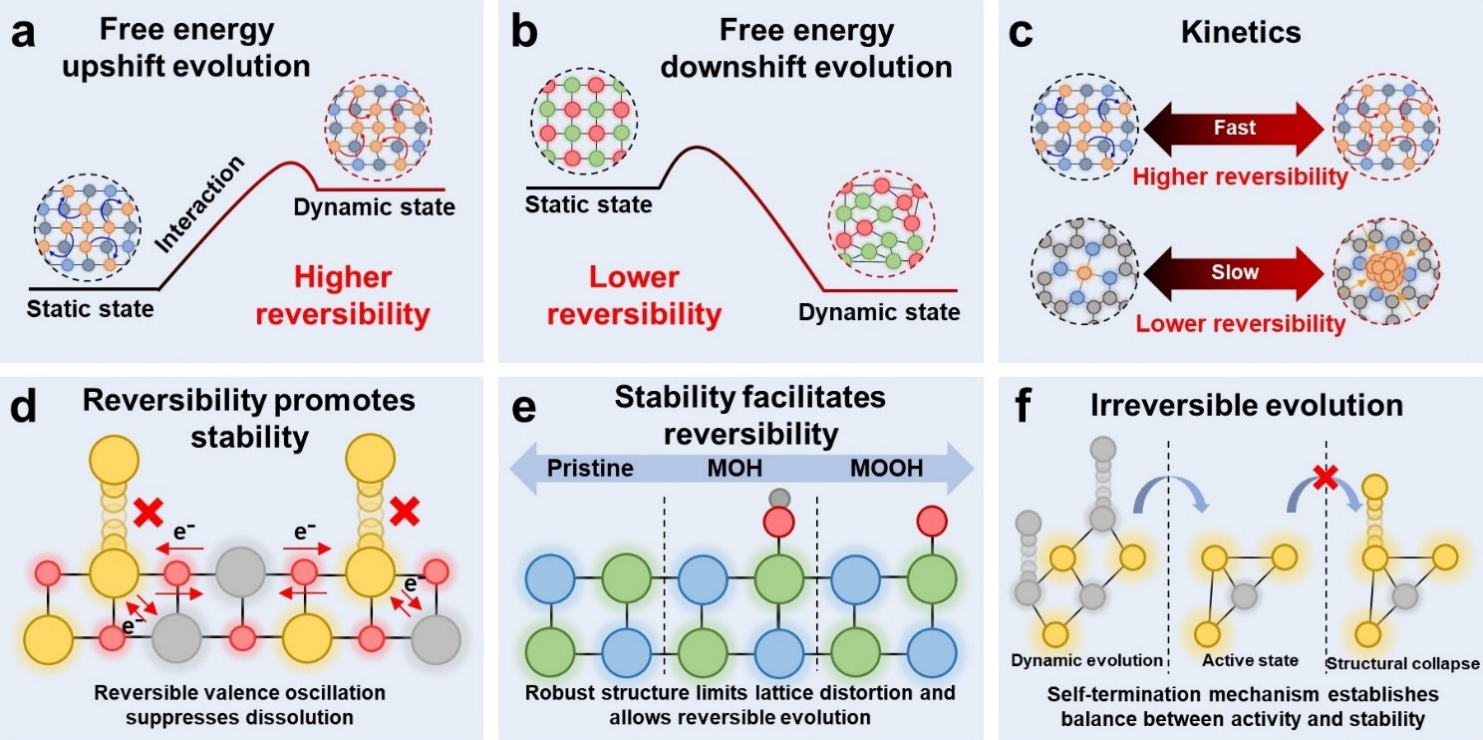
(a and b) Illustration of energy upshift and downshift dynamic evolution, respectively. (c) Kinetics of different dynamic evolutions. (d) Illustration of how reversibility of dynamic evolution promotes stability of dynamic active sites. (e) Illustration of how structural stability facilitates reversibility of dynamic evolutions. (f) Self‐termination mechanism of irreversible dynamic evolution.

The reversibility refers to whether the dynamically evolved active sites can return to their pristine/intermediate structure after the reaction. Thermodynamically, the dynamic evolution process of active sites can be categorized into two types of transformation, the free energy upshift and downshift evolution. According to the Gibbs free energy equation,

ΔG=ΔH-TΔS,

the process with ΔG <0 is spontaneous, while the process with ΔG >0 is nonspontaneous. Under the applied potential and reaction environment, the free energy of the dynamic active sites can be increased during the dynamic evolution as shown in Figure 3a. On the contrary, the applied potential and reaction environment could also activate the dynamic active sites but eventually lead to a free energy downshift evolution as illustrated in Figure 3b. The free energy upshift evolution typically requires the applied potential and reaction environment to maintain the active state of catalysts. Once the applied potential is suspended or reaction environment is withdrawn, the reversed evolution with ΔG <0 becomes a spontaneous process, and the dynamic active sites might return to their pristine structure spontaneously. For instance, the potential‐driven reconstructed Cu nanoparticles were disintegrated and restored to the single atom Cu−N_4_ structure in an ambient atmosphere after electrolysis.^[42]^ Such reversible reconstruction can also be observed on the Cu_1_−CeO_2_ SAC.^[64]^ The electrochemically reconstituted Cu_4_ clusters reversibly transformed back to the single atom Cu_1_−CeO_2_ when the open‐circuit potential was applied. The transformation from metallic to oxidized state is mostly exothermic process with ΔH <0, and the disintegration from nanocluster to single atom is an entropy increase process. Therefore, the above reversed evolutions with ΔH <0 and ΔS >0 are highly spontaneous, and the dynamic active sites are highly reversible. In some cases, the reversed evolution with ΔH <0 and ΔS <0 could be non‐spontaneous since the entropy decrease in atomic level might not be spontaneous. In an energy downshift evolution, the dynamic active sites are transformed into lower free energy state. The reversed evolution with ΔG >0 is mostly nonspontaneous and such dynamic evolution is less reversible. For example, the reconstruction of Fe_0.01_‐Ni&Ni_0.2_Mo_0.8_N to Fe_0.01_&Mo‐NiO underwent the exothermic oxidation of metal and metal nitride, and the entropy‐increase leaching of Mo and N elements.^[71]^ Thus, the reversed process with ΔH >0 and ΔS <0 is nonspontaneous, and such reconstruction is irreversible as reported in their discussion. When the reversed evolution is endothermic, entropy increases, and in general free energy downshift process, theoretically it could be spontaneous conditionally with a certain potential applied or under a certain temperature and the corresponding dynamic evolution could be reversible.

The reversibility of dynamic active sites is further related to the kinetics of reversed evolution. The reversed evolution with fast kinetics suggests a higher reversibility for the dynamic active sites (Figure 3c). The dynamic behavior of active sites will affect the kinetics of their reversed evolution. A more complex chemical state evolution and structure reconstitution of the dynamic evolution can lead to a slower kinetics for the reversed evolution. For example, the simple dynamic evolution of the Cu_1_−CeO_2_ single atom to the Cu_4_−CeO_2_ cluster was reported to be highly reversible.^[64]^ The Cu_4_−CeO_2_ cluster was transformed back to the Cu_1_−CeO_2_ when the applied potential was switched to the open‐circuit potential. In contrast, a more complex restructuring of Cu single atoms to nanoparticles (~5 nm) showed a slower kinetics for reversibility.^[42]^ The restructured Cu nanoparticles did not disintegrated into single atoms immediately when the potential dropped to 0 V vs. RHE. The Cu nanoparticles eventually recovered their single atom Cu‐N_4_ structure after being exposed to the air for 120 days under ambient condition. The transformation of Cu nanoparticle to Cu single atom was more complex and involved more electron transfers than the transformation of Cu cluster to Cu single atom, thus having slower kinetics. A simpler structural reconstitution with less electron transfers typically lead to faster kinetics of the dynamic evolution, thus a higher reversibility.

### 4.2 Stability

During the electrochemical process, stability is closely related to the reversibility of dynamic active sites. Different reversible behaviors can affect the stability of dynamic active sites, and their structural stability can inversely influence their reversibility. According to the above discussion, dynamic evolution can be categorized into reversible and partially irreversible evolution. In this section, the interaction between stability and reversibility is systematically discussed to provide more insights into the stability of dynamic active sites.

A quick reversible evolution can be beneficial to maintain the stable dynamic active phase of electrocatalysts as shown in Figure 3d. Yang et al. reported the valence reduction from Nb^5+^ to Nb^4+^ of Nb_2_O_5_ provided a reservoir for electron and oxygen species, thus inhibiting the oxidation and dissolution of Pt during ORR.^[47]^ The the X‐ray absorption near‐edge structure (XANES) before and after reaction cycles showed that the dynamic evolution of Nb^4+^/Nb^5+^ was reversible. The reversibility of Nb^4+^/Nb^5+^ allowed the Nb_2_O_5_ to consistently prevent the formation of strongly bonded oxygenated species on Pt, which gratifyingly maintained the stability of Pt during oxygen reduction. Jin et al. reported the reversible valence evolution of Re in Re_0.06_Ru_0.94_O_2_ could oxidize the Ru to active state during the on‐site potential, and prevent overoxidation of Ru active sites.^[60]^ The reversible charge transfer between Re and Ru significantly improved the acidic OER activity and stability of Re_0.06_Ru_0.94_O_2_ compared to the pristine RuO_2_. Therefore, the reversible chemical valence evolution can be beneficial to maintain the active state of dynamic active sites and prevent structural destruction during electrocatalysis. On the contrary, a robust structure of electrocatalyst can enhance reversibility of dynamic active sites as illustrated in Figure 3e. Zhao et al. reported a Ni_0.5_Co_0.5_‐MOF‐74 with efficient OER performance in alkaline environment.^[73]^ Although the Ni_0.5_Co_0.5_‐MOF‐74 experienced a complex two‐phase structural transition to Ni_0.5_Co_0.5_(OH)_2_ at low potential then Ni_0.5_Co_0.5_OOH_0.75_ at high potential accompanying with valence oxidation, the robust structure of Ni_0.5_Co_0.5_‐MOF‐74 maintained the uniform distribution of Ni and Co element without destructive separation or aggregation, which gradually recovered its pristine structure after exposed in air for 15 days. Besides, the Cu nanoparticle restored the single atom Cu−N_4_ structure on carbon support after 120 days’ exposure to the air owing to the good stability of N−C structure.^[42]^ Hence, a stable structure of electrocatalyst can limit the structural reconstitution and thus simplify the reversed evolution, which promotes the reversibility of dynamic active sites.

For the irreversible evolution, many of the stable dynamic active sites possess a certain self‐terminating mechanism to stop the dynamic evolution and prevent the structural destruction (Figure 3f).[^68^, ^69^] Wei et al. reported the substitution of Al in La_0.3_Sr_0.7_CoO_3–δ_ facilitated the surface reconstruction into the active CoOOH.^[68]^ Later, the accumulation of oxygen vacancies and lattice‐oxygen oxidation led to the irreversible leaching of Al^3+^, which changed the local electronic structure of the oxides and terminated the reconstruction. Li et al. reported the surface reconstruction of Fe modified Co_2_VO_4_ accelerated by the V leaching.^[69]^ The Fe modified Co_2_VO_4_ was reconstructed into (Fe,V)‐doped CoOOH with excellent OER activity. The residual V in the form of V_2_O_3_ prevented the loss of Co active species and lattice oxygen, which limited the reconstruction to the surface and maintained the bulk structure of Fe modified Co_2_VO_4_. Therefore, the self‐termination mechanism establishes a balance between the dynamic active surface reconstruction and the bulk structural integrity in the irreversible evolution.

## 5 Advanced Techniques to Identify Dynamic Active Sites

**Figure 4:**
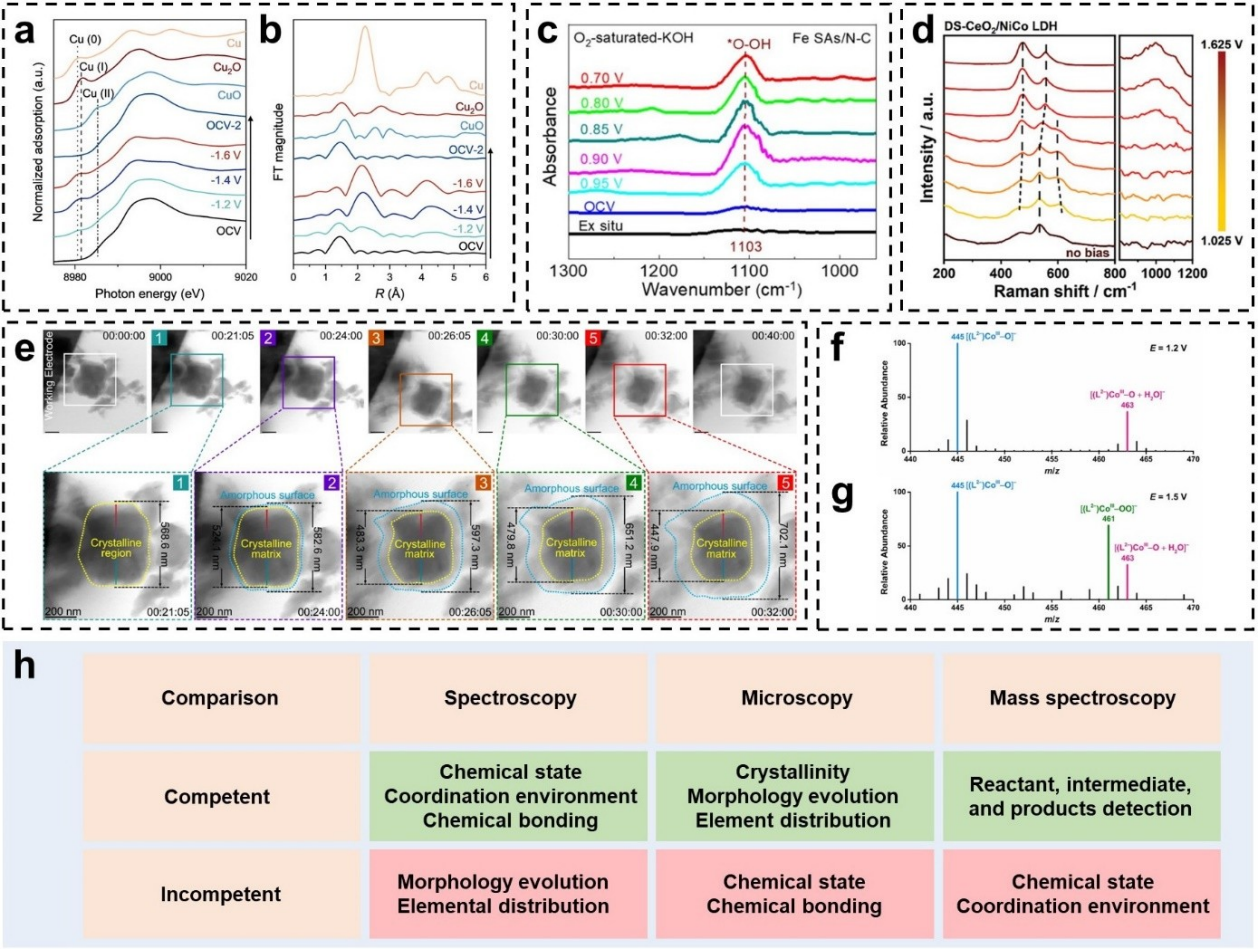
(a and b) *Operando* XANES and EXAFS of Cu_1_‐CeO_2_. Reproduced with permission.^[59]^ Copyright 2023 Wiley‐VCH. (c) In situ FTIR in 960–1300 cm^−1^ range for Fe SAs/N−C. Reproduced with permission.^[73]^ Copyright 2021 American Chemical Society. (d) In situ Raman spectra of DS‐CeO_2_/NiCo LDH. Reproduced with permission.^[74]^ Copyright 2024 Wiley‐VCH. (e) In situ TEM of (NiCo)S_1.33_. Reproduced with permission.^[42]^ Copyright 2023 Nature Publishing Group. (f and g) In situ EC‐MS of cobalt‐oxo and cobalt‐oxo/peroxo intermediates, respectively. Reproduced with permission.^[80]^ Copyright 2022 American Chemical Society. (h) Comparison of different in situ*/*operando characterization techniques.

Recent developments on in situ*/*operando techniques shed light on the intrinsic nature and dynamic evolution of active sites under operation conditions, as well as the in‐depth reaction mechanism, which provides more scientific proof to guide the rational design of catalysts with remarkable activity.[^24^, ^25^, ^74^, ^75^, ^76^, ^77^] In this section, the state‐of‐the‐art in situ*/*operando characterization techniques that characterize the dynamic evolution of active sites are summarized and compared. An overview of schematic of several involved in situ*/*operando techniques is shown in Figure 4.

### 5.1 Spectroscopic Characterization Techniques

Spectroscopic characterizations focus on the evolution of molecular and atomic properties, e.g., chemical states, and electronic structures, and coordination environments of the active sites.[^11^, ^74^] Such information is crucial to understanding the chemical valence evolution and structural reconstitution of dynamic active sites during electrocatalysis.

In situ X‐ray based spectroscopies such as in situ X‐ray photoelectron spectroscopy (XPS) and XAS can be employed to monitor the chemical state and coordination environment changes of dynamic active sites, while the in situ X‐ray diffraction (XRD) can probe the structural changes of dynamic active sites.[^11^, ^75^, ^76^] The in situ optical spectroscopies such as in situ infrared spectroscopy (IR) and Raman spectroscopy offer a potent approach to investigate the chemical bonding and surface adsorbate of dynamic active sites.[^74^, ^78^, ^79^] With the help of in situ*/*operando spectroscopic techniques that often complement electrochemical studies, researchers are now able to directly track the dynamic catalytic processes, identifying reaction intermediates, and revealing the reactive species. The study of dynamic active sites has been benefited from a range of spectroscopies, including XAS, Fourier‐transform infrared (FTIR), and Raman spectroscopy.[^11^, ^74^, ^78^] In particular, operando XAS serves as a unique tool to probe the dynamic behaviors of both the geometric and electronic structure of active sites under electrochemical conditions. XAS can be divided into two absorption regions based on their different origins: the XANES and the extended X‐ray absorption fine structure (EXAFS).^[11]^ XANES gives insight into the oxidation state, electronic structure, and symmetry of the absorbed atom, while EXAFS offers information regarding interatomic distances, coordination numbers, and disorder in the coordination shells.^[11]^ Regarding dynamic active site studies, operando XAS has become a widely used method for monitoring changes in the chemical state and the structural evolution of catalysts under reaction conditions. The dynamic structural evolution of Cu_1_−CeO_2_ had been detected by operando XAS, as the electrochemical reconstitution from copper single atoms (Cu_1_) to clusters (Cu_4_) under reducing potentials, while the reversible transformation of Cu_4_ back to Cu_1_ configurations happened upon switching to the open‐circuit potential (Figure 4a and 4b).^[64]^ Considering that the catalytic dynamic process under scrutiny tends to metamorphosize at multiscale and dimensions in a way, it is highly desirable to integrate complementary techniques to track the same catalyst from different dimensions in real time. For example, the structural dynamic of the atomic active sites within Fe SAs/N−C catalyst during ORR was recently probed by combining synchrotron‐based operando XAS and FTIR.^[80]^ With gradually reducing potentials, operando XAS detected the dynamic release process of coordinated unsaturated Fe−N_2_ moieties and the favorable adsorption of hydroxyl in the form of OH−Fe−N_2_ active sites. Analysis of FTIR data in Figure 4c showed increasing firstly and then decreasing of the *O−OH absorption band, indicating the accumulation and cleavage process of *O−OH intermediate at the HO−Fe−N_2_ active structures. The ceria‐optimized oxygen‐species exchange in NiCo LDH was investigated via the combination of in situ Raman spectroscopy and attenuated total reflection infrared (ATR‐IR).^[81]^ The Raman measurements revealed the double‐shelled (DS−) CeO_2_/NiCo LDH exhibited earlier phase transition at lower potential than the single‐shelled (SS−) NiCo LDH (Figure 4d). And the in situ ATR‐IR analyses suggested the presence of the pre‐adsorption of *OH before OER and the more facile adsorption of oxygen‐containing intermediates during OER for DS‐CeO_2_/NiCo LDH.

### 5.2 Microscopic Characterization Techniques

Microscopic characterization techniques focus on the lattice structure, elemental distribution, and morphology evolution of the electrocatalysts under working environments.[^82^, ^83^, ^84^] In situ*/*operando microscopic can provide localized and time‐resolved information of the electrocatalysts during electrochemical processes, which is highly valuable for deciphering the dynamic behaviors of electrocatalysts.

In situ*/*operando TEM based and AFM based characterizations are two of the most popular microscopic techniques in the study of dynamic active sites.[^82^, ^83^, ^84^] The in situ*/*operando TEM based characterizations can monitor the lattice structure, elemental distribution of electrocatalysts, while the in situ*/*operando AFM based techniques focus more on the surface morphology and electrical conductivity evolution. For example, the in situ TEM measurements in Figure 4e unveiled the time‐expanding amorphous area of (NiCo)S_1.33_ at the pre–catalytic stage of OER.^[46]^ And the in situ atomic high‐angle annular dark field (HAADF) TEM and elemental mapping showed the amorphous evolution and sulfur–oxygen element exchange of (NiCo)S_1.33_ with respect to the increased potentials. Such in situ TEM analyses provide direct and visible evidence of the dynamic lattice and elemental evolution during electrocatalysis. Munz et al. employed the in situ conductive AFM (c–AFM) to investigate the local relationships between electrical conductivity, chemical‐frictional, and morphological properties of the bimetallic Au−Cu catalyst for CO_2_ reduction.^[85]^ The in situ c–AFM revealed the restive CuO_x_ islands corresponding to the local current contrast, and the more conductive convex intragranular region in contrast to the less conductive concave intragranular region of the nanocrystalline Au.

### 5.3 Mass Spectroscopic Characterization Techniques

In situ*/*operando mass spectroscopy can directly detect the intermediates and products at the electrolyte–electrode interface and provide abundant information about the reaction mechanism of the dynamic active sites.[^86^, ^87^] However, the capture of the reaction intermediates on the surface of dynamic active sites remains the biggest challenge for the in situ*/*operando mass spectroscopy. Wang et al. combined the secondary ion mass spectrometry (SIMS) with a vacuum compatible microfluidic electrochemical device to obtain and analyze the secondary ions in the mass spectroscopy.^[86]^ The innovative in situ electrochemistry mass spectroscopy (EC–MS) system was able to capture the short–lived radical intermediate for the first time, and unveil the dynamic evolution of the electrical double layers at the electrolyte–electrode interface during the electro–oxidation of ascorbic acid. Shao et al. combined an ultramicroelectrode (UME) and relay electrospray ionization mass spectrometry to establish the in situ EC–MS.^[87]^ In the EC–MS system, the UME was utilized as micro–EC cell and MS nanospray emitter to drive the reaction and eject the intermediates, then the relay electrospray ionization mass spectrometry detected and analyzed the sprayed intermediates. This EC–MS system has been proved to be highly powerful in capturing the reaction intermediates on a cobalt–tetraamido macrocyclic ligand complex during water oxidation. And for the first time, they captured the short‐live intermediates [(L^2−^)Co^III^−O]^−^ (m/z 445) and [(L^2−^)Co^III^−OO]^−^ (m/z 461) using the in situ EC‐MS (Figure 4f and 4 g).

The above discussions have comprehensively analyzed and exemplified the different advanced in situ*/*operando characterization techniques. Further comparisons of the characterization techniques are presented in Figure 4h. Different characterization techniques can obtain complementary information of the dynamic active sites during the electrochemical reaction, and a rational combination of different techniques can provide a more systematic understanding of their dynamic behaviors.

## 6 Recent Advances of Dynamic Active Site for Electrocatalysis

Electrochemical reduction reactions, e.g. HOR, ORR, HER, OER, CO_2_RR, NRR represent the key research interests in environmental and energy landscapes.[^50^, ^56^, ^88^, ^89^, ^90^, ^91^] For these electrochemical reactions, high‐performance electrocatalysts are of prime importance to boost the energy conversion efficiency. Understanding the dynamic reconstitution of active sites is critical for developing the high‐performance electrocatalysts, as this in situ process generates the real active species that directly affect the adsorption behaviors of the reactants and key intermediates. Moreover, the ever‐changing catalyst–electrolyte interface during dynamic reconstruction fundamentally affects the catalytic activity and durability.

**Figure 5:**
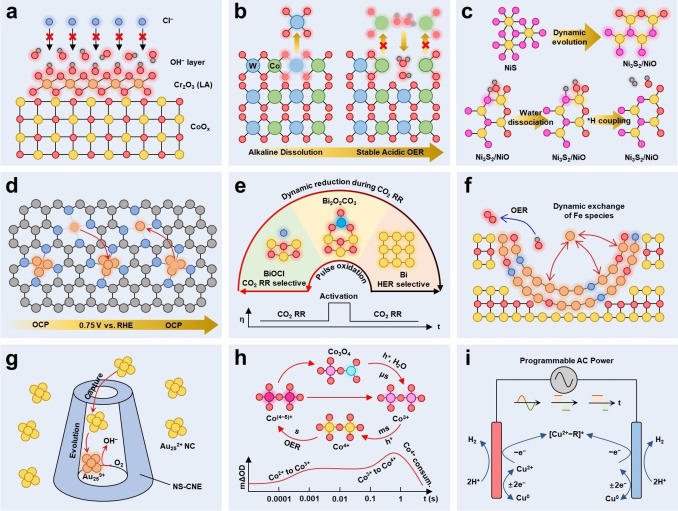
(a) High local alkalinity created by the Lewis acid (Cr_2_O_3_) for seawater electrolysis.^[86]^ (b) Dissolution of W sites and adsorption of H_2_O and OH^−^ for stable acidic oxygen evolution.^[88]^ (c) Dynamic structural reconstruction, valence reduction, and S−O exchange of NiS.^[89]^ (d) Reversible dynamic evolution between CuN_4_/Cu_3_ and CuN_4_/Cu_4_.^[61]^ (e) Reversible transformation between BiOCl and Bi, and the pulse activation for CO_2_RR.^[90]^ (f) Dynamic exchange of Fe species in the nanohole of β‐CoOOH.^[93]^ (g) Cavity nanoelectrode to capture Au_25_ for single‐NP collision electrochemistry study.^[98]^ (h) Transient adsorption spectroscopy for chemical valence state characterizations.^[99]^ (i) Electrosynthesis powered by AC electrical signals.^[100]^

Recently, important progresses have been made for the dynamic active sites in various electrochemical reactions and catalysts. The dynamic evolution of electrocatalysts by the applied potential and reaction environment is ubiquitous but some recent researches discovered the electrocatalysts can conversely change the local environment near the surface of the electrocatalysts.[^92^, ^93^] Lewis acid is an electrophilic species that can strongly adsorb OH^−^ and create local alkaline environment near its surface. Guo et al. developed the Lewis acid–modified Cr_2_O_3_−CoO_x_ electrocatalysts for natural seawater electrolysis.^[92]^ The Lewis acid, Cr_2_O_3_, dynamically split the water molecular and adsorbed the OH^−^ ions, which created a local alkaline environment with pH around 14 near the surface of CoO_x_ (Figure 5a). Hence, the Cr_2_O_3_−CoO_x_ was able to maintain the efficient alkaline performance of CoO_x_ even in natural seawater (pH=7.9). The locally captured OH^−^ layer can repel the attack of Cl^−^ and prevent the precipitation of Mg(OH)_2_ and Ca(OH)_2_, addressing most of the critical challenges of natural seawater electrolysis. Previously, most of the dynamic evolutions of electrocatalyst proceeded under the working condition of that specific reaction, which significantly limited the variation of dynamic active sites. Ram and their colleagues developed a water–hydroxide trapping strategy to stabilize the transition metal element during oxygen evolution under acidic environment.^[94]^ Firstly, the W on the surface of CoWO_4_ (CWO) dissolved as WO_4_^2−^ under the alkaline environment, accompanying with the anion exchange process of WO_4_^2−^→OH^−^ and H_2_O (Figure 5b). The structural delamination and water–hydroxide trapping resulted in the stabilization of the Co ions in the delaminated CWO (CWO–del–48), which then exhibited a stable performance in 0.5 M H_2_SO_4_ over 175 h. When the CWO–del–48 was employed as the OER catalyst, the proton exchange membrane water electrolyzer (PEMWE) delivered 1 A/cm^2^ at 1.77 V and maintained this performance for 608 h, which set up a new elusive benchmark for the Ir– and Ru–free catalysts. Therefore, the dynamic dissolution of WO_4_^2−^ under alkaline environment helped to stabilize the structural integrity of CWO–del–48 in acidic environment, demonstrating a wider possibility of dynamic active sites. Besides, previous research revealed that the dynamic evolved species worked as the direct active sites for the electrochemical reactions. Some latest discoveries showed that the dynamic evolved species might not directly act as the direct active sites but play a significant role in accelerating the critical step of the reaction. For instance, Ding et al. reported the dynamic restructuring of NiS into Ni_3_S_2_/NiO during the cyclic voltammetry (CV) scan (Figure 5c).^[95]^ The restructured NiO bound with OH^−^ to enhance the water dissociation, which is the rate determining step of alkaline HER. With the strong electronic interaction between Ni_3_S_2_ and NiO, the interfacial S sites exhibited modified electronic states and efficient *H coupling property for H_2_ evolution.

The reversibility and stability are critical issues of dynamic active sites and the entanglement between them still remains largely ambiguous.[^2^, ^3^] Recent advances in dynamic active sites provide more in‐depth insights into the relationship between reversibility and stability. The above discussions have shown the interrelationship between reversibility and stability. The recent advances from Ji et al. revealed that the restructuring behavior of Cu species on N‐doped TiO_2_/C (NTC) was jointly related with both the concentration of Cu species and the applied potential.^[65]^ On the Cu_1.5_/NTC, pristine CuN_4_/Cu_3_ was restructured into CuN_4_/Cu_4_ at −0.75 V vs. RHE inside the 0.5 M Na_2_SO_4_ electrolyte with 50 ppm NaNO_3_ as illustrated in Figure 5d. The restructured CuN_4_/Cu_4_ with the closest *d*‐band center to the Fermi level, thus exhibiting excellent activity for direct electrochemical nitrate reduction reaction (NITRR) with the highest NH_3_ yield of 88.2 mmol h^−1^ g_cata_^−1^. Under the more negative potential of −0.85 and −0.95 V vs. RHE, the CuN_4_/Cu_3_ was further restructured into CuN_4_/Cu_7_ and CuN_4_/Cu_9_, respectively, but with suboptimal NITRR activity. Due to the stability of the restructuring Cu cluster, the CuN_4_/Cu_9_ was reversibly restructured back to the CuN_4_/Cu_3_ after exposed in air for 48 h. The high reversibility of dynamic active sites enables the quick evolution between the active and inert state, which can be employed to quickly recover the activity of active sites during the electrochemical process. For example, Liu et al. utilized the highly reversible transition between Bi and BiOCl (BiOCl+2H^+^+3e^−^⇌Bi+Cl^−^+H_2_O, *E* ^*θ*^=0.16 V vs. SHE) to achieve high stability of BiOCl.^[96]^ Their research first disclosed the high activity and selectivity of BiOCl for CO_2_RR, and the high selectivity of Bi for HER. Under the reductive potential of CO_2_RR, BiOCl gradually evolved into metallic Bi, with decreased CO_2_RR activity and selectivity. To retain the desired performance of BiOCl, a pulse chronoamperometry (CA) was developed to oxidize the dynamically reduced Bi and reactivate the BiOCl for CO_2_RR (Figure 5e). At the electrode–electrolyte interface, the dissolution behavior of active sites is critically linked with the stability of electrocatalysts. Such dissolution can be accelerated under anodic potential or harsh environment (acidic, alkaline, etc.), raising significant challenges to the durability of electrocatalysts.[^97^, ^98^] For instance, the Fe species dissolution of Fe based catalyst is an ubiquitous stability issue in the oxidation reactions. Wang et al. discovered the dynamic dissolved Fe was adsorbed more strongly on (1010) edge facets of β‐CoOOH, and the nanohole morphology could slow down the diffusion of Fe species due to the space–confining effect, which together facilitated the redeposition of Fe species within the nanohole of β‐CoOOH as presented in Figure 5f.^[99]^ The highly reversible dissolution/redeposition of Fe species broke the activity–stability trade–off, achieving 10 mA/cm^2^ at a low overpotential of 228 mV and suppressing the Fe leaching by 2 order of magnitude compared to the pure Fe hydroxide catalyst.

To understand the dynamic behavior, reversibility, and stability of dynamic active sites, the development and application of advanced in situ*/*operando characterization techniques are critical. Most of the current in situ*/*operando characterization techniques can only obtain the average information across a large area (compared to a single active site) and a long period (compared to a single reaction step). To further unveil the dynamic behavior of a single active site during a single reaction cycle, such averaging in situ*/*operando characterizations are far from satisfactory, so the localized and ultrafast in situ*/*operando characterizations should be developed.[^100^, ^101^, ^102^, ^103^] Sun et al. employed a nitrogen/sulfur‐functionalized carbon nanoelectrode (NS‐CNE) to capture the Au_25_^2+^ nanocluster (NC) and study its dynamic behavior avoiding the averaging effect (Figure 5g).^[104]^ By isolating a single Au_25_^2+^ NC inside the NS‐CNE, they discovered the dissociation of Au‐ligand bonds and conformation of Au_25_^9+^ NC together depassivated the Au_25_^2+^ NC to the active state. And the occasional reabsorption of the ligand covered the active sites and resulted in the passivation of Au_25_^2+^ NC. Hence, the “ON‐OFF” switches characteristics of Au_25_^2+^ NC can be thoroughly understood with the unique NS‐CNE. Recent advances from Kang et al. deciphered the sequential oxidation of Co_3_O_4_ employing a transient absorption spectroscopy (TAS) in a [Ru(bpy)_3_]^2+^−Na_2_S_2_O_8_−Co_3_O_4_ system.^[105]^ The TAS with μs‐s time scale was able to monitor the ultrafast dynamic evolution of the Co_3_O_4_. As shown in Figure 5h, the TAS measurements discovered the oxidation of the surface Co^2+^ sites to the Co^3+^ intermediates with a time constant of ~736 μs, following by the oxidation of the surface Co^3+^ sites and the Co^3+^ intermediates to the Co^4+^ intermediates with a time constant of ~774 ms, which were then consumed during OER process with a time constant of ~7.1 s. The TAS unveiled the sequential dynamic oxidation of Co sites in Co_3_O_4_ during oxygen evolution, demonstrating the importance of ultrafast in situ*/*operando characterization techniques to the study of dynamic active sites.

Currently, most of the dynamic active site's studies focus on the dynamic response of electrocatalysts towards static electrical input. Recent advances from Lei's group discovered that a dynamic applied electrical signals can also significantly altered the reactivity and selectivity of electrosynthesis reactions on dynamic active sites.^[106]^ A programmed alternating current (pAC) was introduced to systematically adjust the currents, frequencies, and duty ratios. The Cu–catalyzed C−H bond transformation was utilized as a model reaction to study how the programmed waveforms affect the reactivity and selectivity of electrosynthesis reactions (Figure 5i). The C(sp^3^)−H alkynylation reaction between N‐butyl‐2‐oxocyclopentane‐1‐carboxamide and 1‐chloro‐4‐ethynylbenz‐ene was investigated with pAC and direct current (DC). The experimental results showed that the yield of optimized pAC (72 %) significantly outperformed the yield of DC (13 %). The different frequencies, duty ratios, and currents of pAC were proved to play an important role in the reactivity and selectivity of the C(sp^3^)−H alkynylation reaction. Therefore, the pAC protocols with a high degree of freedom hold a great potential for transition‐metal‐catalyzed‐transformation involving electrochemical process.

## 7 Conclusions and Outlook

In this review, we have systematically overviewed the dynamic active sites for electrocatalysis. By comparing dynamic and static active sites, we address that the dynamic evolution of geometric and electronic structures leads to the dynamic catalytic property. The structure‐property correlations of dynamic active sites conclude that the dynamic behaviors prompt superior and versatile catalytic properties. Through analyzing the reversibility from the perspective of thermodynamics and kinetics, we discover not only reversibility can promote stability, but also stability can facilitate the reversibility of dynamic active sites. The rational combination of advanced in situ*/*operando characterizations can monitor the dynamic evolution of electronic structure, coordination environment, geometric structure, morphology change, and reaction intermediates during electrocatalysis, which is the key of unveiling the nature of dynamic active sites. The continuous progress elucidates the rapid development of dynamic active sites, suggesting more endeavors should be dedicated in the field of dynamic active sites. In the following discussion, the critical challenges and opportunities of dynamic active sites are pointed out.

**Figure 6:**
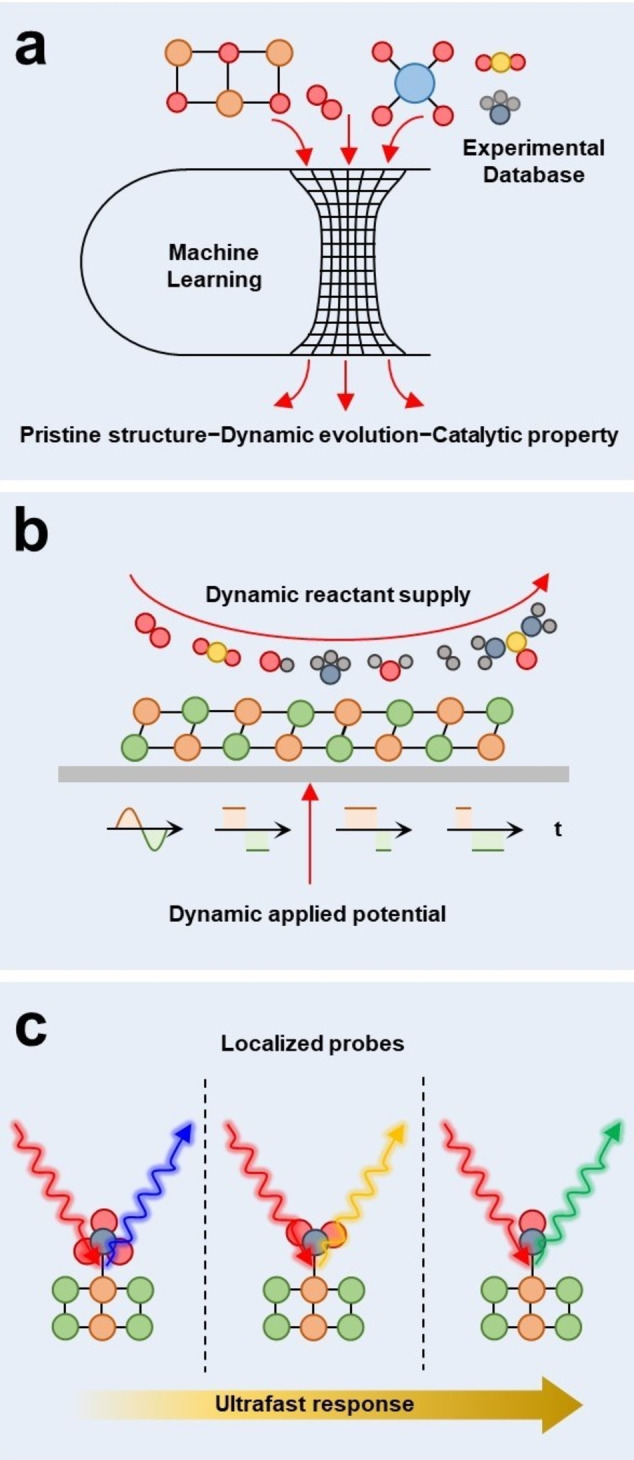
(a) Illustration of machine learning for structure–activity correlation study of dynamic active sites. (b) Diagram of dynamic electrical signal and reactants supplies for dynamic electrocatalysis studies. (c) Illustration of localized and ultrafast in situ*/*operando characterization for dynamic active sites.

*Linking pristine structure, dynamic evolution, and catalytic property*. Previously, tremendous efforts have been made to explore the dynamic evolution behaviors and the activity of dynamic active sites, and a large amount of instances have been studied and reported.[^2^, ^3^, ^12^, ^13^, ^15^] However, most of the studies are isolated researches with different dynamic evolution behavior and mechanism. And researchers are still stuck in the low‐efficiency trial‐and‐error strategy to discover novel dynamic behaviors and efficient dynamic active sites. The general principles governing dynamic behaviors, activity, reversibility, and stability have not been thoroughly analyzed before. Dynamic evolution involves continuous processes and variable parameters, requiring tremendous number of analyses to elucidate the structure‐property correlation. Novel strategies are demanded to unveil the general principles governing the dynamic behavior and catalytic property of dynamic active site. For example, machine learning (ML) is a powerful tool to combine the advanced mathematical algorithm with the empirical experimental database, which has been widely employed to predict and discover new catalysts based on high throughput database. Hence, the large amount of the dynamic active site research can be utilized to train the ML model, which correlates the pristine structure with the reaction environment, the dynamic evolution behavior, and the activity of the real active species as indicated in Figure 6a. Then the ML model can be employed to predict new dynamic active sites with excellent activity, which can greatly accelerate the development of dynamic electrocatalysis. Besides, advanced in situ*/*operando characterization techniques can be utilized to validate the predicted dynamic behaviors, while electrochemical techniques can be used to directly study the electrocatalytic properties of the predicted catalysts. Therefore, ML combined with in situ*/*operando characterization and electrochemical studies can provide an efficient strategy to link the pristine structure, dynamic evolution, and catalytic property together.

*Implementing dynamic electrocatalysis through variable reaction environment*. Most of the previous studies focused on the dynamic evolution of electrocatalysts under static potential and fixed reaction environment.[^2^, ^3^, ^10^] Recent advances from Lei's group have shown the great potential of alternating current signals in the complex electrosynthesis.^[106]^ Moreover, a dynamic reaction environment including the variable supplies of electrolyte and reactant during electrocatalysis can significantly change the catalytic behaviors of dynamic active sites. Especially in the complex organic electrosynthesis process, the multistep nature requires different reactants and applied potentials to drive the reaction. By supplying the correlating dynamic reactant and electrical power rationally as shown in Figure 6b, a fast reaction kinetics can be maintained during the whole reaction cycle and the energy consumption of the electrochemical reactions can be minimized. Therefore, the dynamic alteration of reactants and applied potentials is worth investigating, and its great potential to the complex electrosynthesis should be systemically explored and released.

*Developing localized and ultrafast* in situ*/*operando *techniques*. In situ*/*operando characterization techniques are the ‘eyes’ to observe the dynamic behaviors of electrocatalysts.[^11^, ^24^, ^74^] Previous studies have developed many highly powerful in situ*/*operando characterization techniques including spectroscopic, microscopic, and mass spectroscopic techniques.[^25^, ^75^, ^107^] However, most of the current in situ*/*operando characterizations can only detect the average information across many active sites and over many catalytic cycles. The averaging effect of the current techniques significantly limits the further understanding of dynamic evolution and the structure–activity relationship during electrocatalysis. To address this challenge, localized and ultrafast in situ/operando characterization should be developed to investigate the dynamic behavior of a typical dynamic active site within a single reaction cycle (Figure 6c). In practical, the physical limits of the characterization probes (wave lengths of different electromagnetic waves, scanning rate of electron microscopy, mass transfer efficiency of mass spectroscopy) might pose fundamental obstacles to the localized and ultrafast characterization techniques. Although, the highly scarce active species and the delayed reaction kinetics are not the research interest of developing efficient electrocatalysts, such characteristics can be beneficial for studying the dynamic behavior of a single dynamic active site within a single reaction cycle. The scarce distribution of dynamic active site and slow reaction kinetics lower the technical challenges of the localized and ultrafast characterization.

*Dynamic active sites for chemical industries and beyond*. With the electrification of the modern society, the transition from conventional chemical industry to the energy‐efficient and environmental‐friendly electrochemistry industry is of great interests to the academic and industrial communities. The variable and adaptive catalytic property of dynamic active sites can potentially meet the complex and various requirements from the chemical industry. For example, organic synthesis mostly involves the bonding or cleavage of C−C, C−N, C−S, C−O etc., and multiple complex steps.^[108]^ Hence, the corresponding organic electrosynthesis involves multi‐electron steps, variable and complex reaction intermediates. The hydrogenation reaction, as one of the most frequently used organic synthesis reactions, includes reactant adsorption, active hydrogen formation, surface hydrogenation reaction, and product desorption.^[109]^ Different types of organic hydrogenation reactants such as C≡C, C=C, C−C, C=O, C−Cl/Br/I, etc. have different requirements on electrocatalysts. The dynamic active sites with variable and adaptive catalytic property should be designed to satisfy the various and complex requirements of organic electrosynthesis. Besides, the dynamic and self‐adaptive characteristics of dynamic active sites also hold promising potential to the chlor‐alkali industry, bleach industry, acid production industry, etc.

Briefly, the dynamic active sites are ubiquitous in electrocatalysis, and its adaptive characteristics holds great potential for the next‐generation electrocatalysts. The study on dynamic active sites deciphers the intricate mechanism of structural evolution process and the structure–property relationship, which provides closer insights into the nature of electrocatalysis. Besides, the novel dynamic active sites can potentially lower the utilization of noble metal catalysts in water electrolysis and fuel cell industry, and increase the activity as well as selectivity for the emerging CO_2_ reduction and nitrogen reduction industry. Hopefully, this review can facilitate the establishment of a unified and universal theory for both dynamic and static electrocatalysis, and provide further insights into the development of electrocatalysis‐related industries.

## Conflict of Interests

The authors declare no conflict of interest.

8

## Biographical Information

*Minghui Ning received his B.E. degree from South China Normal University (China) in 2019. He gained his Ph.D. degree of Physics from Department of Physics, University of Houston in 2024. He is currently working at Shenzhen Institute of Advanced Technology Chinese Academy of Sciences for his postdoctoral research. His current research interests focus on the dynamic evolution and reconstruction of active sites for efficient electrochemical reactions*.

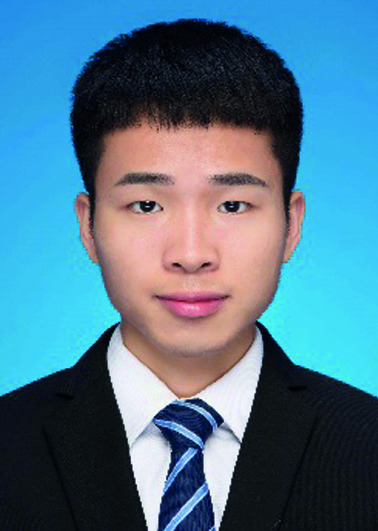

## Biographical Information

*Sangni Wang received her Ph.D. degree from South China University of Technology in 2023. After graduation, she worked as a postdoctoral in Shenzhen Institute of Advanced Technology Chinese Academy of Sciences. Her current research focuses on the fabrication of membrane electrode assemblies and their applications in proton/anion exchange membrane water electrolyzes*.

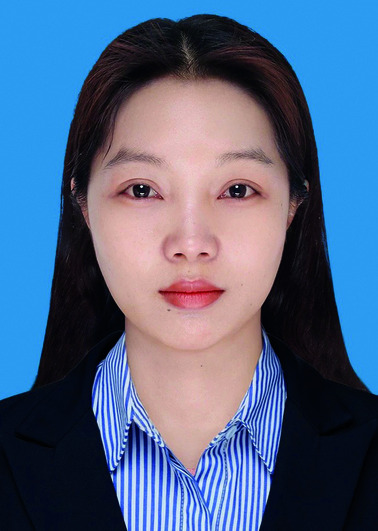

## Biographical Information

*Huanyu Jin is a Professor at the Shenzhen Institute of Advanced Technology, Chinese Academy of Sciences. He completed his Ph.D. in 2020 from The University of Adelaide and subsequently served as a Research Fellow at the Institute for Sustainability, Energy, and Resources at the University of Adelaide. His research focuses on the development of innovative nanomaterials and technologies for renewable energy conversion and electrocatalytic chemical production*.

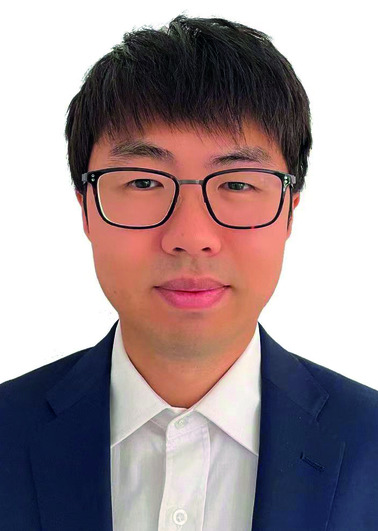

## Biographical Information

*Tianyi Ma is a Distinguished Professor in RMIT University, an Australian Research Council Future Fellow, Fellow of Royal Society of Chemistry, and Clarivate's Global Highly Cited Researcher in both Materials Science and Chemistry fields. He is a leading scientist in renewable energy field, with pioneering work in the areas of functional photocatalytic, electrocatalytic and piezocatalytic materials for renewable energy conversion. He was awarded Australian Acadmey of Science Le Févre Medal and Young Tall Poppy Science Award*.

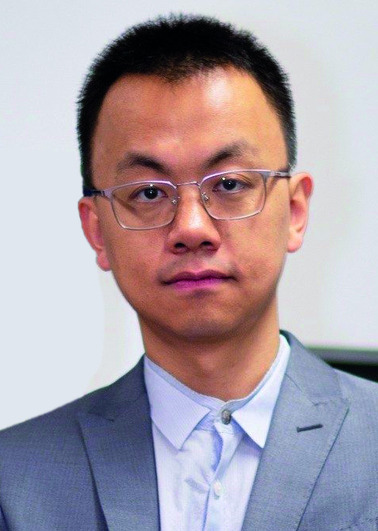

## Data Availability

The data that support the findings of this study are available from the corresponding author upon reasonable request.
